# Resisting top-down anti-genderism: engaging men in feminist social justice

**DOI:** 10.1057/s41599-025-05501-8

**Published:** 2025-07-29

**Authors:** Erman Örsan Yetiş

**Affiliations:** University of Sheffield, Sheffield, UK

**Keywords:** Politics and international relations, Sociology

## Abstract

In Turkey, anti-genderism is notably influenced by top-down politics, which are largely integrated into social engineering within a majoritarian-authoritarian-securitarian political agenda. While grassroots support for this agenda remains limited, it is equally challenging to claim that sweeping resistance from below exists against such politics. Social justice activism based on gender issues can be a common ground for front-line workers, activists, and scholars to resist these politics. In this endeavour, a transformative feminist social justice approach is required that highlights the visibility and autonomy of women’s and LGBTQ+ movements while also incorporating men’s participation. This inclusion is crucial, as top-down anti-gender politics jeopardise these movements’ capability to connect with broader society amidst state-sanctioned violence. Despite growing discontent towards the ruling power’s political agenda, men may struggle to adopt a gender-sensitive perspective and engage in transformative feminist social justice efforts due to their contentious positionalities in the feminist movement. I identify this struggle as a manifestation of slow violence that hinders sociological and political imaginations for an empowering ethical-political stance required for a radical societal transformation. The article explores possibilities of collaborating with men for lasting social transformation toward gender equality and justice, and preventing gendered violence within a feminist framework using the capabilities approach. Benefiting from four studies on gendered violence in Turkey, the article presents novel and robust insights into men’s engagement and proposes the capabilities approach through continuum thinking, emphasising the context of interlinked forms and layered effects of gendered violence alongside ongoing anti-gender politics rooted in masculinist entrenchment. This revealed the challenges male scholars, front-line workers, and activists face in addressing social injustices and violence, highlighting the need for critical reflexivity to overcome these issues. Finally, the article discusses the possible conditions for fostering an environment that can facilitate the cultivation of critical reflexivity for male scholars, front-line workers, activists, and men in general.

## Introduction

“..resisting excessive power is always a complex capacity for humans, one that combines the life-preserving instincts and the capacity to imagine the future.” (Balibar 2020, p. 387).

Currently, anti-gender movements pose a worldwide issue, primarily arguing that the idea of ‘gender’ eliminates the inherent and unavoidable distinctions between men and women (Graff and Korolczuk 2021). This opposition to the so-called gender ideology is inherently contradictory, with the concept of ‘gender’ perceived as dangerous to the extent that it exposes and critiques the existing global gender regime and the power relations established around it. Anti-gender politics pose a serious threat to the rights of women, LGBTQ+ individuals, migrants, refugees, and minorities, who are the most disadvantaged segments of society (Gutiérrez Rodríguez et al. 2018). Beyond preventing these targeted groups from accessing and exercising their rights, anti-gender politics can incite overt acts of violence. Turkey exemplifies the rise of anti-genderism linked to top-down politics, illustrating a different trajectory compared to many Western countries. I contend that anti-gender politics in Turkey are predominantly top-down and are integrated into wider social engineering processes within a majoritarian-authoritarian-securitarian (MAS) political agenda, while grassroots reception remains limited.^1^ Recent studies indicate that there is no overt or prevalent opposition to gender equality in society, or particularly among men (KONDA 2019, 2020; O’Neil and Çarkoğlu 2022; Sancar 2024). Nonetheless, it is equally challenging to claim that these anti-gender politics are met with outright rejection or sweeping public resistance from below, considering that the government has consolidated its political power by gradually putting these politics into practice. Thus, the lack of such sweeping public resistance allows these political climates to persist (Yetiş and Özdüzen 2024; Yetiş 2025a). Moreover, the global rise of anti-genderism, coupled with authoritarian tendencies, fosters similar political climates in different local contexts. Therefore, I assert that gender-based violence (GBV) should also be reconsidered within the contexts where anti-genderism has arisen and subsequently exacerbated it.

The ongoing top-down imposition of anti-genderism in Turkey is characterised by *masculinist entrenchment* (Yetiş and Özdüzen 2024), which acknowledges the deep-rooted and often institutionalised dominance of masculine norms and values, encompassing the structures, practices, and identities that reinforce and perpetuate gender inequality and gendered power dynamics in society and politics. Thus, rather than referring to efforts aimed at reclaiming and reinstating traditional masculine roles and values perceived to have been lost or diminished [as suggested by the concept of masculinist restoration (Kandiyoti 2021; Unal 2021; Kancı et al. 2023)], *masculinist entrenchment* becomes relevant where traditional masculine norms are deeply ingrained and already dominant in the MAS politics. However, it is essential to note that the masculinist entrenchment described here is not exclusive to the gender regime in Turkey^2^, which is also far from being static and unchangeable, harbouring complex paths toward both possibilities for and challenges against gender equality (GE) and justice, as I will continue to unveil its intricacies. I argue that the more nuanced understanding of masculinist entrenchment underpins the top-down anti-gender politics that strategically capitalise on masculinist protection (Young 2003), aligned with the discourses of victimhood and self-preservation of the so-called authentic national identity. This is achieved through the enactment of swashbuckling masculinity, which provides a sense of righteous aggression and violence under the pretext of protecting the family, state, and nation (Yetiş and Özdüzen 2024).

In these circumstances, the MAS political agenda in Turkey creates and sustains polarisation (Arat and Pamuk 2019), which also “defies facile categorisation based on gender since both men and women may find themselves on the opposite side of this divide” (Kandiyoti 2021, p. 215). Thus, as an unintended consequence of this polarisation, social justice activism focusing on gender issues has become a common ground for critical scholars and a broader social movement to combat such political agendas, advocating for egalitarian, social justice-oriented, democratic transformations (Çağatay 2018; Kancı et al. 2023; Özbay and Ipekci 2024; Olcay and Alnıaçık 2025). A transformative approach can strengthen grassroots feminist social justice endeavours by highlighting the visibility, power, and autonomy of women’s and LGBTQ+ movements. This requires building alliances beyond traditional feminist spaces to resist democratic erosion and protect rights (Krizsán and Roggeband 2021). In this regard, it is even more essential to consider men’s engagement in and interactions with these movements, especially since top-down anti-gender politics pose a threat to these initiatives and diminish their ability to connect with broader society through state-sanctioned punitive violence directed at them. While the literature on men’s relationship with anti-genderism primarily focuses on examining men’s role in far-right, conservative and authoritarian populist politics as merely supporters or their alignment with these politics (e.g., Greig 2019; Sauer 2020; Blais 2021; Johanssen 2021; Kaiser 2022; Maricourt and Burrell 2022; Roose and and Cook 2025), I address a significant gap in the literature by contributing to the academic discussion regarding their potential involvement in resisting anti-genderism within these authoritarian populist politics.

On the one hand, I interpret the rise in social violence and ongoing GBV, both occurring with impunity, as a hidden blessing that amplifies feelings of disgust and revulsion toward such violence. Accordingly, state-sanctioned punitive violence, enacted by the MAS political regime in Turkey, reinforces these feelings with a degree of political criticism regarding social injustices (Yetiş and Kolluoğlu 2022). On the other hand, while this growing discontent and criticism may pave the way for a deeper critique of social injustices, it is also likely to be ineffective in promoting meaningful engagement in social justice endeavours (Yetiş and Kolluoğlu 2022; Yetiş 2025b; Yetiş and Özdüzen 2024). Thus, despite the apparent discontent towards the ongoing top-down anti-genderism and MAS political agenda in Turkey, which signifies a potential for men’s engagement in social justice endeavours, it is crucial to recognise that this will not occur straightforwardly, as men may find it challenging to adopt a feminist and gender-sensitive perspective due to their contentious positions within the feminist movement and their engagement with it (Yetiş and Kolluoğlu 2022).

This article deploys *continuum thinking* (Boyle 2024) by utilising the analytical concept of *slow violence*^3^ (Nixon 2013), which refers to attritional yet hidden harms that accumulate over time and space. This distinct analytical perspective enables us to illuminate the links between overtly aggressive and repulsive forms of violence and harms (e.g. state-sanctioned punitive violence) primarily evident in the practices of the MAS political regime in Turkey and more hidden, unrecognised forms of violence that get in the ways for resisting this ‘violence regime’ (Hearn et al. 2022).^4^ I identify *fatalistic normalisation, daunted managerialism*, and *afflictive condemnation* as manifestations of slow violence that hinder sociological and political imaginations for the development of an ethical-political stance required for a radical societal transformation (Yetiş and Bakırlıoğlu 2023) and, thus, these must be acknowledged and addressed by male scholars, activists and front-line workers in their respective fields and practices. *Fatalistic normalisation* involves the active production of ignorance to prevent awareness, inducing a sense of learned helplessness and resignation. In contrast, *daunted managerialism* involves the postponement of awareness in the form of cruel optimism, which harbours a pinch of hope for gradual improvement both in the present and in the future, thereby preventing wider sociological and empowering political imaginations. Even when a degree of political awareness is achieved and embraced in society, we can observe that *afflictive condemnation* co-opts such awareness, diverting it from a broader understanding of and commitment to social justice. While I developed these concepts in a previous study (Yetiş and Bakırlıoğlu 2023), I reintroduce them here in the service of resisting anti-genderism, as they reveal and ‘name’ what inhibits the sociological and political imaginations necessary for men’s engagement in social justice.

The analytical perspective built on these concepts can contribute to *critical reflexivity*, stimulating cognitive and emotional awareness for social transformation, and more effectively resist top-down anti-genderism. On this basis, the article primarily aims to explore various possibilities for collaborating with men in pursuit of enduring societal transformation for social justice, GE, and the prevention of GBV in alignment with a feminist social justice-oriented framework through the *capabilities approach*. This approach can foster the necessary sociological and political imagination as well as alternative empowering visions with a robust political-ethical stance to resist top-down anti-gender politics and authoritarian social policies, highlighting how male scholars, front-line workers, and activists can assume the role of active participants and facilitators within this framework.^5^ I believe Turkey presents a distinctive context characterised by ongoing top-down anti-genderism, and this manuscript examines the barriers and opportunities both within and beyond this context to address the globally escalating phenomenon of anti-genderism.

In pursuit of this aim, this paper benefits from my research on Turkey, including (1) men’s perceptions, thoughts and experiences of gendered violence (Yetiş 2019), (2) the psychosocial approach to interviewing men on gender-based violence (Yetiş 2020), (3) male front-line workers’ challenges and opportunities in addressing men’s violence (Yetiş and Kolluoğlu 2022), and (4) top-down anti-genderism with masculinist entrenchment in Turkey (Yetiş and Özdüzen 2024). Through a re-analysis of these studies using various theoretical concepts with an interpretive approach (Heaton 2004; Köhler et al. 2025), this manuscript aims to provide a robust theoretical contribution to men’s engagement in resisting top-down anti-genderism in pursuit of feminist social justice. Hence, the arguments are not presented solely on the empirical data from these four studies, which have already been published elsewhere. Instead, I develop novel insights into men’s engagement in societal transformation towards feminist social justice in a context marked by the MAS political agenda (i.e., Turkey). Firstly, I elaborate on paternalistic social politics and two different approaches to social justice —liberal and transformative— relevant to the context in which anti-gender politics predominate. Then, I present the capabilities approach concerning GBV prevention by expanding its utilisation through *continuum thinking* to comprehend the *conducive context* that enables a range of interlinked forms and multilayered repercussions of GBV, where anti-gender politics within masculinist entrenchment prevails. I discuss the barriers men, in general, face in developing alternative imaginations for a political-ethical stance that can facilitate social justice endeavours committed to non-violence. This contributes to an examination of the challenges that many male scholars, front-line workers, and activists encounter when addressing issues of social injustices and violence, emphasising the importance of adopting *critical reflexivity* (Burrell and Flood 2019) by following continuum thinking as a key component for overcoming these challenges. Ultimately, through this approach, I identify and discuss possible conditions that can foster an environment conducive to cultivating this reflexive mindset among male scholars, frontline workers, activists, and men in general.

As a male scholar and activist, I situate my research and advocacy efforts within feminist scholarship, believing that my analysis can deepen our understanding of how to counter the current trends of the globally pervasive phenomenon of anti-genderism, which may be complicated by its locally diverse manifestations. This understanding is a prerequisite for developing more effective feminist strategies and methods of resistance and transformation. Grasping social justice through a feminist lens is crucial for effective activism, advocacy, and social research. In this way, collaborative studies and initiatives that aim to encourage men’s participation in women’s empowerment and GE can also reckon with feminist political-ethical dilemmas related to men’s ambivalent positionalities in their research and activism (Ruxton 2020). This can be particularly effective when male scholars, front-line workers, and activists take on the role of active participants and facilitators in feminist justice endeavours. Such initiatives will enhance the growth and dissemination of feminist perspectives on social justice.

## Towards a feminist and transformative approach to social justice

Before we can establish a feminist understanding of social justice, it is important to question what social justice can mean in the context where top-down anti-gender politics prevail under paternalistic social politics. Social justice here calls for a re-consideration from various perspectives, including liberal and transformative approaches, in the face of this paternalistic framework and its associated social injustices. We need to elaborate on the distinctions between the liberal and transformative approaches to examine their reverberations on anti-gender politics thriving under the paternalistic framework. In this section, I employ the concepts of *fatalistic normalisation* and *daunted managerialism* (Yetiş and Bakırlıoğlu 2023) to illustrate how the paternalistic framework underpins a *conducive context* as a generative mechanism that enables a range of interlinked forms and multilayered repercussions of GBV. The liberal approach to justice, as an alternative response to such a paternalistic framework, however, remains inefficient since it falls short of having a meaningful, transformative, and empowering potential for both individuals and communities.

The paternalistic framework is evident in Turkey, rooted in masculinist protection embedded in conservative neoliberal social policies and welfare chauvinism promoted by the MAS political agenda of the incumbent government (Yetiş and Özdüzen 2024). This framework is mostly concerned with practical and urgent needs while compromising long-term strategic objectives and empowerment plans and involves a top-down approach in which those in power make decisions on behalf of the whole society, often assuming they know what is best for people and, thus, can lead to a lack of agency and autonomy for those affected by these politics. It promotes *fatalistic normalisation*, referring to the acceptance of systemic issues as unchangeable or inevitable (Yetiş and Bakırlıoğlu 2023). This can manifest as a belief that oppressed groups need guidance and control from those in power, reinforcing the status quo and hindering efforts to address the root causes of inequality. Such a belief can also lead to *daunted managerialism*, characterised by a bureaucratic and/or charity-based approach to managing social issues, often focused on short-term efficiency and control rather than social transformation (Yetiş and Bakırlıoğlu 2023). A paternalistic framework can lead to policies that manage rather than thoroughly address social problems, perpetuating systemic inequalities while seeking to maintain loyalty to the status quo. In Turkey, the paternalistic framework is adopted and entrenched by the ruling power, aligning with the masculinist entrenchment in the MAS political agenda (Yetiş and Özdüzen 2024). This demonstrates that political support and approval are often pursued through loyalty and gratitude (Akkan 2018). The shift in social politics to prioritising social services for families at the expense of women’s rights is evident in the renaming of the Ministry of Women and Family to the Ministry of Family and Social Services. The Directorate of Religious Affairs has adopted the conservative agenda of the political power (Yilmaz and Albayrak 2022), which influences women’s lives and family dynamics. Its role in spreading anti-gender politics includes promoting traditional female roles by encouraging submission and discouraging divorce, even in cases of domestic abuse. This institution receives significant resources to offer counselling primarily for families and women, while other institutions’ support services (e.g. social work services) are sidelined (Karakaş 2022). In addition, as the nation grapples with financial instability due to soaring inflation, alongside political unrest caused by the unlawful detainment of dissident politicians and journalists (Yetiş 2025c), President Erdoğan has revealed his intention to propose a new constitutional referendum aimed at safeguarding family values from what he describes as homosexual propaganda (Al-Ali et al. 2025), declaring 2025 the Year of the Family (Kamadan 2025). This announcement reflects the government’s conservative, familial social policies in line with an anti-gender political agenda.

On the other hand, upon withdrawal from the Convention on Preventing and Combating Violence against Women and Domestic Violence (widely known as the Istanbul Convention) in 2021 and rising impunity surrounding GBV, calls for a liberal social justice approach emerged as a viable alternative to the paternalistic framework (Yetiş and Kolluoğlu 2022). This is primarily due to its focus on policies and legal reforms that offer swift solutions amidst the erosion of the rule of law driven by the current top-down anti-genderism efforts. However, the liberal approach suggests a rather individualistic and one-size-fits-all perspective, addressing only predetermined needs to achieve equal opportunities and processual capabilities within the existing social order (Capeheart and Milovanovic 2020). In a liberal approach, *fatalistic normalisation* emerges as the perception of legal and policy activism as the final recourse, which unwittingly undercuts grassroots social transformation, given the increasing scepticism towards resistance and an overestimation of bottom-up political support for ongoing anti-genderism (Yetiş and Özdüzen 2024), which also inadvertently entrenches resignation and learned helplessness. The liberal approach may also fall into *daunted managerialism*, where the emphasis on top-down policy and legal reforms only leads to a bureaucratic approach that prioritises procedural fairness over substantive justice. Beyond this, since the rule of law has deteriorated over the last two decades of the ruling power (Cengiz 2020), this approach can hardly help us see through how the legal and policy instruments have been either gradually hollowed out or bent in favour of the MAS political agenda. Of course, the legal or policy activism arising against Turkey’s paternalistic framework is not solely rooted in a liberal approach; nonetheless, it fails to adequately tackle anti-genderism due to its lack of a transformative agenda. Thus, this sort of activism tends to anchor around managing the disquieting consequences of a persistent ‘violence regime*’* (Hearn et al. 2022) rather than addressing its root causes.

Transformative social justice, however, promises a more comprehensive understanding of justice that can call forth societal change by going beyond the generative mechanisms underlying the existing injustices and paving the way for empowerment not only for individuals but also for collectives (Capeheart and Milovanovic 2020). Creating and maintaining a space for various actions and options, as well as imaginations that are not predetermined or imposed by existing mechanisms, is essential for a transformative approach. As we reflect on what we can do individually and collectively, a transformative understanding of social justice emerges as an active manifestation of empowerment in our everyday lives to resist social injustices. Rather than viewing vulnerabilities, sufferings, and incapabilities as inherent traits of individuals, cultures, and systems, we should scrutinise the systems and environments that constantly reproduce these vulnerabilities, sufferings, and incapabilities, along with the relationships established within these frameworks. All these factors—structures, situations, and connections—help us understand the causes behind ongoing injustices.

A transformative approach can strengthen the feminist social justice framework by highlighting the visibility, power, and autonomy of women’s and LGBTQ+ movements. However, it is also essential to consider men’s engagement in and interactions with these movements, especially since top-down anti-gender politics pose a threat to these initiatives and diminish their ability to connect with broader society.^6^ This illustrates how political decisions and actions related to gender issues are underpinned by a punitive mechanism created and maintained by political power. The primary strategy here is to position LGBTQ+ individuals and women’s rights advocates against an imagined conservative family model that allegedly upholds society’s core values, thereby accusing these groups of undermining it. As a result, political power can utilise extra-legal measures to demonise LGBTQ+ activists and certain feminist groups by isolating their political struggles from one another and other oppositional factions (Zengin 2024). This is not limited to Turkey but is also relevant to other contexts in which anti-genderism with authoritarian tendencies becomes conspicuous (Yetiş and Özdüzen 2024). More recently, for example, President Trump’s anti-DEI (i.e., diversity, equality, inclusion) rhetoric and its widespread top-down adoption in the US (Boso 2025) are likely to impact other local contexts as well, precipitating the attacks against women and LGBTQ movements, ethnic/racial minorities and immigrants and cutting their ties with broader society. Thus, the potential engagement of men should strengthen the connection between the feminist social justice movement and broader society, empowering collective social action against these anti-gender politics. To this end, it is essential to understand the significance of the women’s and LGBTQ+ movements and their struggles for rights, both in the past and today. Such engagement must connect these rights to specific real-world problems and struggles rather than leaving them as abstract principles. Nonetheless, men may find it challenging to adopt such a feminist and gender-sensitive perspective due to their controversial positions in the feminist movement and to engage with it. To ensure men’s involvement in feminist social justice endeavours, alliances should be forged where women and LGBTQ+ individuals hold key positions in the socio-political realm. Additionally, men must nurture alternative imaginations that both convey their interests and, beyond their interests, align with political-ethical stances supporting these endeavours.

Certain feminist groups might be wary of men’s participation in feminist movement due to political and ethical issues. These issues pertain to the potential control exerted by male scholars and activists, which could undermine the movement’s autonomy (Brown and Ismail 2019). There are additional concerns about the redistribution and further fragmentation of already scarce resources, particularly for research and interventions on women’s empowerment and GE, if these resources are also allocated to efforts focused on men’s engagement (Orme et al. 2000). While these concerns are accurate in some respects, they still raise at least three issues: Firstly, lack of men’s engagement can reinforce the identification of gender as a ‘women’s issue’, ignoring that men are gendered too (Nayak and Suchland 2006). This also unwittingly leads to the exclusion of not just heterosexual cisgender men but also gay and transgender men, dismissing their gender-related needs (such as sexual health), concerns (including sexual harassment, discrimination and social exclusion based on their gender identity, expression and sexual orientation) and their demands for inclusive empowerment on the basis of these gender issues (Graaff 2021). Secondly, it hinders men from fully examining how a gender-specific issue like violence impacts their own lives (Reinicke 2022), limiting their perception of potential perpetrators against possible female victims only as ‘other’ men (mostly as a marginalised group, as poor, immigrant and minority) and ignoring men’s positions as victims or witnesses of such violence (Yetiş 2019). Thirdly, the focus on empowerment and capability within gender issues is narrowly framed as solely related to women’s gains and accomplishments, creating an impression that men are not required to engage with these matters. This perspective can also buttress a perception of GE as a zero-sum game (Messner 2016; Yetiş 2019, 2020), suggesting that women’s empowerment undermines men’s. Consequently, this can result in men either feeling indifferent to gender issues or perceiving themselves as potential victims of GE. These dynamics can also be interpreted as slow violence that directly or indirectly bolsters existing anti-genderism within masculinist entrenchment.

Grasping social justice through a feminist lens is vital for activism and advocacy. Collaborative studies and initiatives that encourage men’s participation in women’s empowerment and gender equality can tackle feminist political-ethical dilemmas. This can be particularly effective when male scholars, front-line workers, and activists take on the role of active participants and facilitators in feminist justice endeavours. Such efforts will enhance the growth and sharing of feminist perspectives on social justice.

## Capabilities and continuum thinking for gender-based violence

Feminist transformative social justice is not a goal to be achieved; rather, it indicates an unending transformative process in which we can orient ourselves within existing social circumstances. It does not occur in a straightforward manner either; it requires social action alongside a political programme and the development of a set of concepts that align with the programme. The capabilities approach is both a part of this conceptual set and a compass for the development of other concepts and methods to entrench such social justice endeavours by providing the means to resist mechanisms that produce violence.

The capability approach critiques traditional developmental policies, arguing that economic growth cannot resolve social injustices on its own (Sen 1980; Nussbaum 2011; Capeheart and Milovanovic 2020; Gangas 2020). This approach bases human capability on solving urgent problems and eliminating injustices resulting from structural inequality as soon as possible. It adopts a pluralistic and non-reductionist understanding of human development, which contrasts with abstract concepts of nation and society. This approach offers a concrete and contextual understanding of human development and needs (Eguia Huerta 2017). When considering social justice within the framework of this understanding of capabilities, we must ask ourselves what we can do for each individual and community (Pereira 2013). Realistically identifying and resolving obstacles to capabilities can only be achieved in this manner.

Violence here can be regarded as anything that impedes the realisation of capabilities. By this definition, we can expand its meaning and make it much more comprehensive, interpretable, and politically charged. Following this logic, gender inequality can be seen as the main reason for impeding the capabilities of women, which also paves the way for different forms of GBV. However, as Walby et al. (2017) cautioned us, there is a risk of taking gender inequality as the most overarching form of GBV. This can lead to an overdetermination that tends to dilute the very definition of violence without differentiating between its various forms and the repercussions it can generate. On the other hand, we also need to shy away from the very siloed definitions of and piecemeal responses to gendered violence since they can be counterproductive to understanding and resisting such violence occurring and transposing through different ramifications and dimensions within the social structure. Here, in alignment with the capabilities approach, *continuum thinking* enables us to comprehend and confront interconnected forms of violence without considering them as equivalent or analogous.

*Continuum thinking* in relation to GBV draws on Liz Kelly’s (1987) conceptualisation of the continuum of violence against women, signifying multiple factors at play behind such ongoing violence beyond individual, interpersonal and stand-alone incidents of violence. These factors mainly include the culture of everyday sexism, women’s poverty and economic dependence, gender pay and pension gaps, unequal participation and representation in political life, unequal access to public services and common goods, and sexist stereotyping in the media (Kelly 1987). As an analytical framework, *continuum thinking* makes it possible to distinguish between different forms of violence while revealing links between them, considers violence as existing on a spectrum from subtle forms of harassment to severe physical violence and helps understand how seemingly minor acts of aggression can escalate into more severe forms of violence (Boyle 2024). It also highlights the normalisation of certain behaviours, enabling a culture of violence as a *conducive context* (Kelly 2016). Here, we can define top-down anti-gender politics and masculinist entrenchment in Turkey as the *conducive context* that enables both GBV with varied forms and a ‘violence regime’ with impunity. Furthermore, we can assert that globally rising anti-genderism, coupled with authoritarian tendencies, further exacerbates the conditions in local contexts. Thus, contemporary politics in the global arena can also be seen as the broader *conducive context* in terms of *continuum thinking* while recognising both the contextual differences and the continuum between them.

Unlike narrow and stand-alone or over-encompassing and undifferentiated definitions of violence, continuum thinking helps us to apply the concept of capability in relation to a *conducive context* that enables a wide range of violence. The absence of violence, dangers, and threats is a crucial prerequisite for gaining access to and reaping the benefits of numerous other rights as well as cultivating political-ethical responsibilities for others’ wellbeing (Yetiş and Kolluoğlu 2022). When we consider barriers to capabilities in such a context in alignment with the concept of slow violence, we can also recognise the most hidden aspects of violence-generating mechanisms begetting attritional harms that accumulate over time and space. Hence, gender inequality and injustice, like all other inequalities and injustices, can be defined as a violence-generating mechanism in such a context to the extent that it constitutes an obstacle to capabilities. In this regard, the capabilities approach emphasises addressing the root causes of violence and enhancing individuals’ and communities’ capabilities to create environments where people have the freedom and opportunities to live without fear of violence. Having said that, we still cannot assume that all forms of GBV are equivalent under the umbrella concept of gender inequality, especially regarding their contextually and positionally differentiated causes and effects.

Similarly, *continuum thinking* provides a more comprehensive, intersectional, and inclusive understanding of GBV with different dimensions and forms, which goes beyond the binary thinking of men’s violence against women. Even though GBV is mostly conceptualised as violence against women, *continuum thinking* critically expands its definition, including violence against LGBTQ+ and gender non-conforming individuals, violence directed towards men and boys, violence between men, as well as the ongoing militaristic culture and practices both in times of conflict and during everyday life in so-called peaceful times (Cockburn 2010; Graaff 2021; Boyle 2024). Following *continuum thinking*, we not only embrace a more comprehensive understanding of GBV but can also attain insight into how the different forms of social violence and injustices are imbued with GBV in a *conducive context* of anti-gender politics and how men are involved in these. Upon developing such insight, it becomes more possible to contemplate how men can engage in social action, resisting anti-genderism within authoritarian populist politics.

When integrated with the capabilities approach, *continuum thinking* requires emphasising the importance of agency and its empowerment. By recognising the broader spectrum of violence, we can develop a more nuanced understanding of advocacy and interventions in pursuit of social justice that can operate in line with empowering individuals to challenge and resist violence at all levels. Enhancing capabilities ensures that individuals have the necessary resources and support to exercise their agency and live free from violence (Yetiş and Kolluoğlu 2022). Once again, this underlines the importance of understanding experiences of violence and how capabilities are influenced by intersectional and compounding factors (Crenshaw 1991) such as gender, ethnicity, age, class, and sexuality, as well as being bodily or mentally abled/disabled. This helps in developing more inclusive and effective interventions that address the diverse needs and potential of individuals and communities. However, the empowerment perspective, building on capabilities, is not limited to the potential of utilising or attaining various resources to exercise agency; it also involves cultivating alternative imaginations to develop a political-ethical stance (Pease 2022) that can pave the way for social justice endeavours to commit to non-violence. We first need to understand what is getting in the way of cultivating such imaginations and then ponder how we can create an environment that makes this cultivation possible.

To address this, I will explore the obstacles faced by male scholars, front-line workers, and activists as they tackle issues related to social injustice and violence, highlighting the vital role of *critical reflexivity* in overcoming these challenges. Moreover, I will outline the necessary conditions for creating an environment that can facilitate such cultivation for these scholars, workers, activists, and men in general. As noted in the introduction, dissatisfaction with the prevailing top-down anti-genderism and MAS political agenda in Turkey, rooted in *masculinist entrenchment*, harbours the potential for men to engage in social justice endeavours. However, it is crucial to understand that this engagement will not happen in a straightforward manner. To embrace alternative empowering visions for a feminist social justice framework, it is essential for male scholars, front-line workers, and activists to cultivate an ethical-political stance that can exceed their current capacities through critically addressing *fatalistic normalisation, daunted managerialism* and *afflictive condemnation* embedded in their practices and perspectives. Thus, I hope this discussion will facilitate the realisation of this potential by presenting transformative pathways.

## Going beyond capacity: active engagement of men in feminist social justice endeavours

I emphasised the importance of understanding social justice from a feminist perspective for activism and advocacy. Joint research and programmes that promote men’s involvement in women’s empowerment and GE can address feminist political and ethical challenges by drawing on the insights provided by feminist literature and critical studies on men and masculinities. This approach is especially impactful when male scholars, front-line workers, and activists engage as active participants and facilitators in the pursuit of feminist justice endeavours. However, to achieve this, we must first highlight the distinction between capacity and capability, as there is a tendency to conflate these two terms. Capacity refers to the actual and ongoing functioning within existing circumstances and structures (Trevithick 2012). Since the current capacities constitute an operational ground for building capabilities, properly apprehending them should be the first step for assessing the opportunities and challenges faced by scholars, front-line workers and activists. Nevertheless, it still offers a limited perspective for fostering the development of transformative and empowering capabilities. The capacities encompass the powers and duties outlined in existing policies and practices within the restrictive framework of a *conducive context*. These include what can be done with the means at our hands. However, when capacities are severed from capabilities, *fatalistic normalisation*, *daunted managerialism*, and afflictive *condemnation*, in the form of slow violence, arise. *Fatalistic normalisation* involves the acceptance of the current order and the ways in which it is operationalised, as evident in the paternalistic framework and liberal approach to social justice. Such a normalisation eventually begets a resignation in the form of disbelief in change. The obstacles created by social, political, cultural and economic structures, as well as institutional culture in which scholars, front-line workers and activists operate, are fatalistically accepted if not totally endorsed. For instance, the fact that the Higher Education Authority revoked its policy on gender equality by condemning the very concept of ‘gender’ as inappropriate to societal norms and values (Uçan Çubukçu 2021), aimed at inhibiting gender-sensitive research and advocacy agenda in universities, is likely to strengthen *fatalistic normalisation* among scholars who shy away from adopting a gender perspective in their studies lest they are punished by the incumbent political power.

Different from *fatalistic normalisation*, *daunted managerialism* is pursued by a cruel optimism that involves the logic of ‘making do with’ these structurally encompassing problems and doing the best we can within our capacity in the face of ongoing top-down anti-gender politics and conservative, authoritarian social policies. For instance, neoliberal and conservative social policies, in conjunction with the growing influence of the Directorate of Religious Affairs and Islamist charity organisations that organise around political allegiance to the ruling power, undermine the empowerment framework of social services founded on rights and needs (Yetiş and Özdüzen 2024). Consequently, front-line workers are increasingly frustrated by their limited capacities in their roles. Yet, they also acquiesce to a diminished legal and policy landscape that corresponds to a limited array of individual managerial manoeuvres, providing palliative, partial, and short-term interventions and solutions, all based on a fragile trust in relative improvement within such a framework (Yetiş and Kolluoğlu 2022). Against *fatalistic normalisation* and *daunted managerialism*, the capabilities need to be strengthened by sociological and political imaginations that are potentially transformative for achieving radical social action to address these seemingly tenacious structural problems.

Capabilities, here, refer to institutional and collective capabilities rather than individual competitors in a competitive field. Thus, we should regard capabilities as resourceful potentials to be continually developed for collective empowerment, rather than merely a compilation of qualifications to be individually attained. As a core part of societal transformation in favour of a feminist social justice framework, the empowerment of individuals and communities remains essential; yet, the empowerment of scholars, front-line workers and activists themselves is equally important, and nurturing *critical reflexivity* in pursuit of sociological and political imaginations is key to accomplishing this. However, in parallel with the increasing circulation of the term’ *critical reflexivity’* in scientific studies, as well as practice-based interventions and activist actions through social movements, the inattentive and tokenistic reception of the term, which often takes the shape of an egocentric and self-indulgent manner, has become problematic (Ryan 2024). This problematic reception is partially reflected through a judgmental performance of virtue-signalling in the form of *afflictive condemnation* (Yetiş and Bakırlıoğlu 2023) by externalising gendered violence as other men’s problem or as an overarching systemic issue and as part of other problems generated by political power and their conservative policies (Yetiş and Kolluoğlu 2022). This problematic reception also draws on a condemnation of others who are regarded as having a lesser degree of socio-political awareness or lacking self-awareness of their personal experiences during research, intervention practices, and activism. Male front-line participants in my study generally presume that their interest in participating in GBV research indicates their greater openness to reflexivity compared to other men who do not participate in such research (Yetiş and Kolluoğlu 2022). Although such a presumption has some merit in terms of accuracy, considering the prevalence of indifference and reluctance among male front-line workers and practitioners regarding their lack of participation, this statement reveals less about participants’ reflection on their interests and aspirations for actively working on this issue. Instead, they continue to strive to uphold their virtuous position couched in persistent pessimism, at the cost of feeling isolated and incapable of making any significant changes in their field. To avoid such inconsequential adoption of the term as a benchmark for competitiveness or individualistic virtue, which also resonates with masculinist values in stoic and heroic styles, our best bet is to appeal to Bourdieu’s conceptualisation of *critical reflexivity*. It is essential to address directly (the logic or operationalisation of) the field that shapes and reproduces the very embodied practices, positionalities, and relationships blended with various motivations, interests, and aspirations. As argued by Bob Pease (2022, p. 225), “we need to engage in processes that will challenge the institutionalisation of privilege within political, economic, religious and educational systems.” Dominant groups use ruling relations to regulate subordinate groups. Primarily, altering the ways of participation in the relationships within the field (Burawoy 2019), based on such reflexivity, can also influence the broader structure.

A capacity-oriented perspective deprived of sociological and political imaginations also risks reinforcing existing gendered norms and stereotypes, lining up with masculinist protection to address the problems we encounter in the field of our expertise. Developing the capabilities of scholars, front-line workers, and activists requires a holistic and comprehensive approach, taking into account a variety of factors, including gendered institutional norms, practices, and various subjective positionalities. Heuristically reverting to the existing orientations within the current capacities, already infused with masculine norms and practices within masculinist entrenchment, is a common occurrence, particularly when imagining alternative ways to reorient their practices towards novel directions and solutions becomes challenging (Yetiş and Bakırlıoğlu 2024). To be effective in the field, front-line workers, activists and scholars need to be strengthened in many aspects to go beyond their existing capacities by enhancing their capabilities. Firstly, it is necessary to nourish *critical reflexivity* through a continuous and reliable supportive environment, which provides open space for critical scrutiny without being judgmental.

Robust critical reflexivity, which exceeds the capacity, can initially emerge from the very oppression experienced by male front-line workers, activists, and scholars. Experiences such as being a victim or witness of domestic violence and abuse during childhood and youth can prompt critical questioning of the detrimental effects and impairments caused by these experiences, both in their own lives and in those of others. Several front-line worker participants (Yetiş and Kolluoğlu 2022), for example, specifically highlighted how such experiences significantly influenced their career choices in front-line work, aiming to be part of the solutions to problems they are already familiar with. Besides individual experiences, my studies with men (Yetiş 2019, 2020; Yetiş and Kolluoğlu 2022) also illustrate that living under oppressive and authoritarian socio-political conditions with the intensification of social injustices under the *conducive context* of top-down anti-gender politics (socio-economic deprivation and political misrepresentation of marginalised communities of poor, minorities and immigrants), state brutality via police violence targeting politically dissident groups, and prevalent arbitrariness of social violence with increasing impunity, all of which cause moral injuries, can instigate their critical thinking on such oppressive situations. As a result, this can also harbour a potential for their engagement in correcting such injustices to some extent, in pursuit of the *continuum thinking* on the connection between various forms of violence, both in their own lives and in the lives of others, as well as in the broader society. Thus, *continuum thinking* can facilitate sociological and political imagination in resisting top-down politics rather than resigning to or ineffectively condemning them.

The second aspect I would like to address to enhance capabilities is the struggle to transform stereotypical gender roles and responsibilities embedded in men’s practices within a *conducive context* that enables GBV where top-down anti-genderism prevails. As ‘caregiving’ becomes naturalised as part of women’s labour through their perception, men assume ‘protective’ roles that align with their understanding of masculine gender roles. In my studies (Yetiş and Kolluoğlu 2022) and other relevant studies (e.g., Khunou et al. 2012), men perceive ‘caring for someone’ as a responsibility reserved solely for women, while they regard ‘taking care of someone’, which implies protection or patronisation, as a role more suited to men. Here, taking care of someone also encompasses mental engagement rather than emotional or physical intimacy (Trevithick 2012). Due to its emphasis on reason and decision-making, ‘taking care’ is considered a predominantly masculine activity. Such distinction, for example, clearly illustrates the gendered nature of front-line work in social services. The portrayal of labour requiring emotional and physical intimacy as women’s work, contrasted with labour demanding mental authority and decision-making as men’s work, not only perpetuates gendered stereotypes within the front-line professions but also contributes to gender inequality within the field itself. In this way, the undervaluation of care and emotional labour is *fatalistically normalised*.

Accordingly, there is a need for men to question the ethical values and qualities associated with these gendered roles in their work and practices. By adopting a protective and controlling role, male front-line workers, scholars, and activists reproduce and reinforce gendered stereotypes, division of labour, roles, and responsibilities inherent in their practices (Yetiş and Kolluoğlu 2022). Additionally, this impedes the implementation of feminist values and principles in the development of practices that support GE. Consequently, in alignment with conservative social politics, the interventions concerning the prevention of GBV are reduced to only addressing the urgent practical needs of women as protection from immediate violence of aggressive men, neglecting long-term strategic interests aimed at empowering women. The fact that male front-line workers, scholars and activists define their roles and responsibilities to prevent GBV around masculine protectionist or benevolent sexist attitudes perpetuates the existing gender regime based on the exaltation of masculine understanding of power (Yetiş 2020), which enables GBV, let alone prevent it. In this context, in order to combat violence more effectively, training programmes on GBV prevention should be restructured to overcome gendered moral values that fall into the duality of ‘care’ and ‘protection’ (Trevithick 2012).

In line with enhancing capabilities, preventing GBV and resisting top-down anti-gender politics requires additional training programmes and support groups focused on gender awareness in their practices. These programmes should not only focus on the relationships between front-line workers and their recipients or researchers and their participants, but also empower these workers and researchers to reflect on their own roles and authority in relation to their gendered subjectivities and positionalities. The training in these programmes should promote openness and provide concrete examples that enable these men to connect their experiences with the dynamics of internalised oppression and domination (Yetiş 2020). In this respect, it is essential to review and re-position the gender perspectives and knowledge informing their practices in accordance with critical and transformative approaches. For male front-line workers, scholars, and activists, these approaches are vital for comprehending their own positionalities and for gaining a deeper understanding of gender issues, not solely based on some abstract norms or principles perfunctorily adopted to condemn violence but also through a *critically reflexive* inquiry, which they should incorporate into their training and practice from a relational perspective within a *continuum thinking* on violence.

Lastly, it is essential to discuss the significance of men actively participating in and facilitating an environment (Pease 2022) that encourages them to examine their interests and investments in their own masculinity and develop capabilities (beyond their current capacities) for political-ethical responsibility, accountability, and action. To achieve this, the perception of GE as a zero-sum game, where women gain and men lose, must be dismantled. With the dissolution of this perception, a legitimate public space may emerge for men to reflect on the role of GBV in their own lives, along with its various forms and multi-layered harms and costs. Such public environments should be established as platforms free from discrimination, oppression, and prejudice (Flood 2019; Pease 2022; Stewart et al. 2023), thereby providing reassurance while also fostering debate that enables men to critically examine their interests, investments, and privileges in terms of *continuum thinking*. All public and civil society actors working with men bear the responsibility to cultivate such an environment. Particularly in the *conducive context* featured by top-down anti-genderism, all forms of engagement with men should discourage the establishment of masculine cooperation (Katz 2025) amongst men, ensuring that there is no space for challenge or provocation that could obstruct transformation and collaborative action aiming at feminist social justice. It will be challenging for front-line workers, scholars, and activists to navigate this delicate balance. For them to recognise and uphold such an ethical stance, it is crucial that they continuously question their own masculinity before, during, and after their work and practices. Professional discussion groups and supervisory support need to be developed to facilitate this self-inquiry.

## Conclusion

This article argues that Turkey represents a unique context of top-down anti-genderism and explores the associated challenges and opportunities to strengthen resistance against gender-based violence in its various interconnected forms within such a context. Anti-genderism within the majoritarian-authoritarian-securitarian political agenda in Turkey fosters polarisation that transcends simple gender categorisations, placing both men and women in opposing camps. In the face of this, social justice activism unites critical scholars with a broader movement to counter these political agendas, advocating for egalitarian and democratic transformation. By adopting a transformative approach, the feminist social justice framework can be strengthened, enhancing the visibility, empowerment, and autonomy of women and LGBTQ+ individuals. As top-down anti-gender politics threaten women and LGBTQ+ movements and weaken their societal ties through increasing punitive violence, men’s engagement with them becomes even more crucial. In Turkey, the rise of impunity in social violence, especially gender-based violence, increases society’s revulsion toward such acts. Additionally, state-sanctioned violence by the current regime intensifies these feelings and incites a political critique of social injustices. While this critique can potentially lead to a deeper exploration of these issues, it often fails to inspire genuine engagement in feminist social justice. Thus, even though there is discontent regarding Turkey’s top-down anti-genderism and authoritarian political agenda, which suggests potential for men’s involvement in social justice endeavours, realising this will be challenging. Men may struggle to embrace a feminist and gender-sensitive lens due to their intricate positionality within the feminist movement and their commitment to it. This article primarily aimed to explore various strategies for collaborating with men in the pursuit of sustainable societal change, promoting social justice, gender equality, and the prevention of gender-based violence. It examined the pathways leading toward a feminist social justice framework through the capabilities approach, which can cultivate the necessary sociological and political imaginations, as well as alternative, empowering visions, with a robust political-ethical stance to resist top-down anti-genderism couched in authoritarian populist politics.

Through a critical discussion on liberal and transformative approaches to social justice, particularly in relation to anti-gender politics under paternalistic social policies, the article advocates for a comprehensive and transformative stance. It introduces the capabilities approach to address gender-based violence using continuum thinking to grasp the interconnected forms and repercussions of such violence amid anti-gender politics within masculinist entrenchment. Continuum thinking is deployed through the analytical concepts of *fatalistic normalisation*, *daunted managerialism* and *afflictive condemnation* as manifestations of slow violence, which hinder men from envisioning alternative, non-violent political-ethical positions in favour of social justice. The article highlights the challenges faced by male scholars, frontline workers, and activists in addressing social injustices, emphasising *the importance of critical reflexivity* in overcoming these challenges. As a core aspect of societal transformation, empowering individuals and communities remains essential; however, empowering scholars, front-line workers, and activists is equally vital. Nurturing *critical reflexivity* in the pursuit of sociological and political imagination is crucial to achieving this. Finally, the necessary conditions for creating supportive environments are outlined, which can foster the development of the capabilities, beyond existing capacities, of male scholars, front-line workers and activists in alignment with feminist perspectives, thereby contributing to a deeper understanding of the current drives towards the globally prevalent phenomenon of anti-genderism. The manuscript calls for scrutinising interlinked forms of violence through the lenses of social injustice and cultural structures involving gender norms and stereotypes, advocating for the acknowledgement of men’s roles in perpetuating them.

This understanding constitutes a prerequisite for developing more effective feminist strategies and methodologies for broader grassroots resistance and transformation. Here, comprehending social justice through a feminist lens is essential for activism and advocacy that aims to open a space for men’s engagement. Following the capabilities approach with continuum thinking, collaborative studies and initiatives can promote the active participation of men in gender issues, women’s empowerment and gender equality, reckoning with feminist political-ethical dilemmas. This approach may prove particularly effective when male scholars, front-line workers, and activists engage as active participants and facilitators in feminist justice endeavours resisting top-down anti-genderism. Such undertakings will foster the growth and dissemination of feminist perspectives regarding social justice.

## Data Availability

The datasets generated during and/or analysed during the current study are not publicly available in order to protect participant privacy and due to the sensitive nature of the topic. The data might be available from the corresponding author on reasonable request.

## Footnotes

1 In Turkey, political power reinforces its authoritarian hegemony by allowing varying degrees of violence from non-state actors, as various societal actors gain power by supporting the state’s coercive authority. The anti-rights front, consisting of fundamentalist religious groups, ultra-nationalists, feudal networks, mafia-like organisations, and state-sponsored NGOs, aligns with the majoritarian-authoritarian-securitarian political agenda and reinforces masculinist power dynamics through cultural intimacies between political power and themselves. This front should not be viewed as grassroots reactionaries autonomously forming anti-gender movements. Instead, the heterogeneous and contradictory amalgamation of Turkey’s anti-rights front highlights a strategic collaboration rooted in cultural intimacies. However, top-down anti-gender politics fuel masculinity-in-defence, invoking victimisation and self-preservation narratives that rationalise violence. This can bolster reactionary movements, including racist, homophobic, and misogynistic organisations beyond the political authorities’ control (Author et al. 2024a).

2 While this article mainly focuses on Turkey, the concept of masculinist entrenchment can also be valuable for analysing top-down anti-genderism in other contexts that share authoritarian or semi-authoritarian characteristics, such as Russia, Eastern Europe, and certain regions of the Global South.

3 Nixon (2013, p. 2) defines slow violence as “a violence that occurs gradually and out of sight, a violence of delayed destruction that is dispersed across time and space, an attritional violence that is typically not viewed as violence at all”.

4 The concept of ‘violence regime’ recognises that different forms of violence, such as gender-based violence, intimate partner violence, and anti-LGBTQ+ violence, are interconnected and often reinforce each other and emphasise the importance of understanding the structural factors that contribute to violence. The concept involves both the material and discursive aspects of violence and proposes examining the physical manifestations of violence as well as the ways in which violence is represented and understood in society. This includes examining how power dynamics, social norms, and institutional practices shape and sustain violence.

5 As a note, this article does not present a detailed account of women’s and LGBTQ movements in Turkey or an assessment of their impact across society, but rather focuses on the barriers and opportunities for men actively engaging in transformative feminist social justice endeavours. I am not specifically focusing on self-proclaimed ‘pro-feminist’ men either, as they represent a relatively small number of people among male scholars, front-line workers and activists and they lack a persistent organisational structure that can impact the social justice endeavours. Beyond these, I find addressing the ambivalent positionalities of men within these feminist social justice endeavours more pressing regarding the opportunities and limitations.

6 Rights-based civil society organisations pursuing social justice sometimes face exclusion and criminalisation due to their activities contradicting the conservative policies of political power, exemplified by attempts to shut down the Platform to Stop Femicide *[Kadın Cinayetlerini Durduracağız Plaformu]* (Tahincioğlu 2022).
